# Investigation of Multifunctionality of Electrospun Poly(vinyl alcohol) Nanofiber Membranes Incorporating Boric Acid and Nanoencapsulated Curcumin: Filtration Performance, Antibacterial Activity, and Environmental Impact

**DOI:** 10.1021/acsomega.5c03317

**Published:** 2025-06-10

**Authors:** Sepideh Keyvani, Farideh Golbabaei, Rasoul Esmaeely Neisiany, Mohammad Reza Pourmand, Saba Kalantary, Oisik Das, Abbas Rahimi Foroushani, Ensieh Masoorian, Saied Goodarzi

**Affiliations:** † Department of Occupational Health, School of Public Health, 48439 Tehran University of Medical Sciences, Tehran 1417613151, Iran; ‡ Department of Polymer Engineering, 49569 Hakim Sabzevari University, Sabzevar 9617976487, Iran; § Biotechnology Centre, Silesian University of Technology, Krzywoustego 8, Gliwice 44-100, Poland; ∥ Department of Pathobiology, School of Public Health, 48507 Tehran University of Medical Sciences, Tehran 1417613151, Iran; ⊥ Department of Civil, Environmental and Natural Resources Engineering, 5185 Luleå University of Technology, Luleå 97187, Sweden; # Department of Epidemiology and Biostatistics, School of Public Health, 48507 Tehran University of Medical Sciences, Tehran 1417613151, Iran; ∇ Medicinal Plants Research Center, School of Pharmacy, 108842 Tehran University of Medical Sciences, Tehran 1417613151, Iran

## Abstract

Nanofibrous structures have a wide range of applications, including air filtration for fine particles. This study fabricated bead-free nanofiber membranes from aqueous poly­(vinyl alcohol) (PVA) and PVA/boric acid (BA) solutions containing varying concentrations of nanoencapsulated curcumin (CUR) using the electrospinning method to investigate filtration performance against aerosols and bioaerosols, antibacterial activity, mechanical properties, and biodegradability. The prepared membranes were morphologically examined using field emission scanning electron microscopy (FE-SEM). In addition, Fourier transform infrared spectroscopy (FTIR) was used to investigate the incorporation of CUR into the fibers. The higher CUR content resulted in thicker fibers and significantly improved the mechanical properties of the samples. The filtration performance of the bead-free samples was evaluated for different particle sizes (0.3–3 μm) at airflow rates of 28.3 and 32 L min^–1^. The incorporation of CUR enhanced filtration performance against chemical particles and microorganisms, mechanical characteristics, as well as antibacterial activity in the nanofibers. Nanofibers containing CUR could provide superior filtration performance, achieving approximately 100% efficiency for 0.3 μm particles and a pressure drop of 167 Pa at 32 L min^–1^. Also, lower CUR concentrations in the nanofiber membranes demonstrated high bacterial filtration efficiency (BFE) (above 96%) against Staphylococcus aureus (S. aureus). Nanofibers containing CUR showed more effective antibacterial activity against Escherichia coli (E. coli) compared to S. aureus. Furthermore, biodegradability, as a specific aspect of environmental impact, was investigated in PVA and PVA/BA nanofibers containing CUR. According to the results, the biodegradability rate of PVA nanofibers decreased as CUR and BA were added to the membrane structure. A greater CUR concentration could also increase the biodegradability rate of PVA/CUR and PVA/BA/CUR nanofibers. As a result, green electrospun nanofibers containing CUR and BA offer a promising solution for environmentally friendly air filters targeting chemical particles and microbial contaminants.

## Graphical abstract

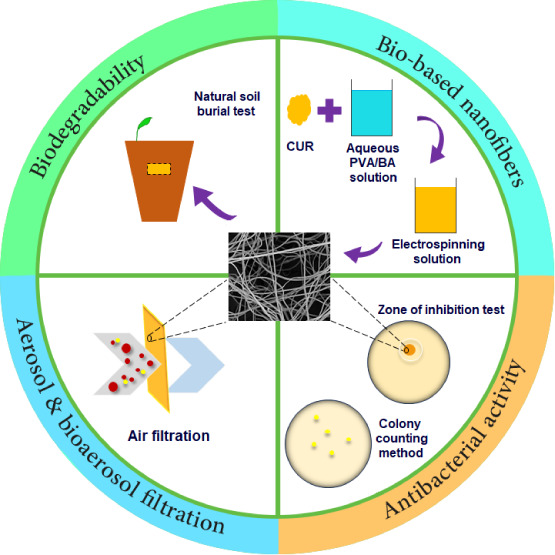

## Introduction

The rapid industrialization globally is causing environmental problems including particulate pollution that endangers human life and public health.^1^ Particulate matter (PMs) are produced by industrial processes,^2^ transportation, stationary combustion, and natural sources.^3^ It is classified into three types, including PM_10_ (particles with an aerodynamic diameter less than 10 μm), PM_2.5_ (particles with an aerodynamic diameter less than 2.5 μm),^4^ and PM_1.0_ (particles with an aerodynamic diameter less than 1.0 μm).^5^ Suspended PM in the air, or aerosols, may get into the lungs and bronchi, causing respiratory^6^ and cardiovascular diseases,^7^ as well as mental disorders, such as depression, anxiety, bipolar disorder, psychosis, and suicide.^8^ Furthermore, tiny particles (such as PM_1.0_) have a higher risk of entering the human lungs and skin, causing more damage.^9^ As a result, it is critical to control air pollutants through filtration, which is the most common control strategy.

Electrospinning, among other techniques,^10^ is a low-cost method for fabricating air filters that applies a high voltage to a polymer solution, consequently forming nanofibers,^11^ ranging from several nm to less than 1000 nm in diameter.^5^ According to the literature review, nanofiber membrane filters fabricated using the electrospinning are gaining popularity recently due to their facile handling^5,12^ and nanofiber properties such as smaller diameter, larger specific surface area, higher porosity, and homogeneity^13^ controllable morphology of fibers^14^ via regulation of common process parameters such as polymer concentration, voltage, feed rate, and needle-collector distance.^15^ Furthermore, electrospun nanofiber membranes can provide effective filtration performance (compared to conventional filters) in terms of high filtration efficiency and minimal pressure drop due to their aforementioned structural properties.^16^

Petroleum-based polymers, such as polyethylene and polypropylene, are the primary source of traditional filtering materials.^17^ However, these aforementioned polymers are not biodegradable and thus have the potential to generate harmful microplastics in the environment. Therefore, it is preferable to use biodegradable polymers to reduce plastic waste that ends up in the environment.^18^ PVA is a low-cost polymer with many hydroxyl groups and properties such as being nontoxic to the human body, biodegradable, biocompatible, and containing hydrophilic functional groups, making it soluble in water.^19,20^ As a result, the use of eco-friendly electrospun membranes made from PVA can mitigate both environmental and human health consequences associated with the use of nonbiodegradable polymers.

Antibacterial filters can capture airborne pollutants and help prevent respiratory infectious diseases caused by bioaerosols,^21^ which account for around 24% of atmospheric particles and 5–10% of total suspended particulate mass.^22^ The biological pollutants, which include airborne microorganisms, e.g., bacteria, can attach to PMs and gradually enter human organs via the respiratory system, resulting in health issues, such as cardiovascular and respiratory diseases.^21,23^ To counter the above-mentioned issues, antibacterial PVA-based nanofibers have been developed using metal nanoparticles,^18,24,25^ graphene oxide nanosheets,^26^ and other materials (e.g., benzalkonium chloride, quaternary ammonium salt, and fucoidan).^27–30^ In this study, CUR was used as an antibacterial agent in the precursor polymer solution. Curcumin ((1*E*,6*E*)-1,7-bis­(4-hydroxy-3-methoxyphenyl)-hepta-1,6-diene-3,5-dione) is one of the major active ingredients of turmeric extract, which is derived from the root of Curcuma longa,^31^ and is one of the very few offering antimicrobial agents of plant origin.^32^ Curcumin shows a wide range of pharmacological activities, including anticancer,^33^ anti-inflammatory,^34^ antioxidant,^35^ antiparasitic properties,^36^ and neuroprotective activities,^37^ as well as antibacterial effects against both Gram-negative, e.g., E. coli and Gram-positive bacteria, e.g., S. aureus.^38,39^ Despite its aforementioned advantages, curcumin has limited water solubility. Thus, nanoformulation of curcumin has emerged, such as CUR, to address this problem.^40,41^

BA is a Lewis acid with a p*K*_a_ of 8.92–9.24 that is affected by temperature, ionic strength, and concentration, and generates hydronium ions (H_3_O^+^) and borate ions (B­(OH)_4_^–^) in aqueous solutions.^42,43^ BA was also discovered to be an effective constituent in electrospun nanofiber membranes, improving both filtration performance and fire safety.^44^ However, no previous studies have been conducted to hybridize BA and CUR within electrospun PVA nanofiber membranes. Furthermore, no research has investigated the BFE of a PVA electrospun nanofiber membrane containing BA and CUR, adding novelty to this study. Totally, in this research, bead-free electrospun nanofiber membranes were produced using the electrospinning technique, which involved biobased materials and water as a green solvent. In addition, the electrospun membrane’s multifunctionality was studied in terms of filtration performance, BFE, antibacterial activity, and biodegradability.

## Materials and Methods

### Materials

PVA (72 kDa, hydrolyzation ≥98%) and BA were obtained from Merck Co., Germany. CUR powder, branded as CuroMax, was kindly provided by NANOAGE Co., Iran. Sodium chloride (NaCl), Müller-Hinton agar (MHA), tryptic soy agar, and Müller-Hinton broth, tryptic soy agar (TSA), and tryptic soy broth (TSB) were obtained from Merck Co., Germany. All of the solutions were made with deionized water. Furthermore, all materials and chemicals were utilized without further purification steps.

### Preparation of the Solutions

The homogeneous PVA and PVA/BA solutions were prepared using a previously reported method.^44^ Briefly, a solution of PVA was prepared at a concentration of 6 wt %. In addition, the 5.5 wt % PVA/3 wt % BA solution was created by thoroughly mixing the prepared BA solution with the prepared PVA solution.

CUR concentrations ranging from 5 to 15 wt % were added separately to each of the PVA and PVA/BA solutions and thoroughly stirred to produce homogeneous antibacterial solutions. Table 1 presents the resulting compositions.

**Table 1:** Sample Codes and Electrospinning Parameters of the Nanofiber Membranes

Codes	PVA (wt %)	BA (wt %)	CUR (wt %)	Voltage (kV)	Distance (cm)	Feed rate (mL h^–1^)
N-1	6	–	5	15	18	0.75
N-2	6	–	10	15	18	0.75
N-3	6	–	15	15	18	0.75
C-1	6	3	5	15	18	0.50
C-2	6	3	10	15	18	0.50
C-3	6	3	15	15	18	0.50

### Fabrication of the Electrospun Membranes

The nanofiber membranes were prepared with an electrospinning machine (Fanavaran Nano-Meghyas, ESDP30, Iran). The solutions were poured into a 3 mL plastic syringe containing an 18-gauge steel needle. Then, the nanofibers produced under the electrospinning parameters listed in Table 1 were collected on a commercial substrate layer of spunbond polypropylene (around 17 g m^–2^ basis weight, 100 μm thickness, Baftineh Co., Iran) with a drum collector rotating at 170 rpm at room temperature, and relative humidity of approximately 28 ± 1%.

### Morphological and Chemical Characterizations

The morphology of the prepared membranes was investigated using FE-SEM (TE-SCAN, MIRA III, Czech Republic) at a voltage of 30 kV after coating the samples with a thin layer of gold. The nanofibers were assessed using the ImageJ software. The average nanofiber diameter was then calculated by randomly evaluating 50 nanofibers. Furthermore, the porosity of the electrospun membranes was determined using a method described in the preceding study.^45^

Furthermore, the functional groups of the nanofiber membranes were identified using Fourier transform infrared spectroscopy (FTIR; Thermo, AVATAR, USA) in the IR region 4000–400 cm^–1^.

### Mechanical Test

The mechanical test was performed on the prepared membranes similarly to the preceding research.^44^ To summarize, the thickness of various points on each membrane was determined using a Schopper-type thickness gauge (Ogawa Seiki Co., Japan). Subsequently, tensile properties of the samples were measured using a uniaxial tensile machine (Instron, 5566, USA) equipped with a 50 N load cell and a crosshead speed of 5 mm min^–1^, in accordance with the ASTM D3039.^46^ The experiments were conducted three times to calculate the mean and standard deviation (SD) of the values.

### Filtration Performance Testing

The filtration performance of nanofiber membranes was measured using a media test machine (Fanavaran Nano-Meghyas, Iran). Each filtration performance test included measuring the PM filtration efficiency and pressure drop for each membrane over a period of 5 min. The filtration efficiency (difference in PM concentrations before and after the membrane) was measured for neutralized NaCl particles ranging in size from 0.3 to 3 μm. The air pressure drop was measured in membranes for airflow rates of 28.3 L min^–1^ (∼4.7 cm s^–1^ surface velocity),^47^ and 32 L min^–1^ (∼5.3 cm s^–1^ surface velocity).^48^ All the PM tests were repeated three times for each membrane. To evaluate filtration performance comprehensively, the quality factor was determined using eq 1.^49^

1 $$\mathrm{Quality factor}=-\frac{\ln\left(1-\mathrm{Filtration efficiency}\right)}{\mathrm{Pressure drop}}$$

### BFE Testing

The BFE of the fabricated nanofiber membranes was determined using a modified method based on the ASTM F2101-19 standard,^50^ which involves comparing the filter’s upstream and downstream bacterial counts. The suspension of S. aureus ATCC 6536 in TSB was aerosolized by a nebulizer with a mean particle size (MPS) of 3.0 ± 0.3 μm and delivered to the test membrane (100 mm × 100 mm) in an Anderson sampler at a flow rate of 28.3 L min^–1^. The bacterial challenge was maintained at 1.7–3.0 × 10 ^3^ colony-forming unit (CFU) mL^–1^. A TSA plate was placed beneath the membrane specimen and then incubated at 37 °C for 24 h. Then, the number of colonies was counted. The test was also conducted without a membrane to determine the number of colonies exposed to the sampler as a positive control. BFE was determined using the colony counting method (eq 2). Each bacterial test was run three times on each sample to determine the mean and SD of the colony count.

2 $$\mathrm{BFE}=\frac{\left(\mathrm{Mean colonies for control sample}-\mathrm{Mean colonies for test sample}\right)}{\mathrm{Mean colonies for control sample}}\times 100$$

### Evaluation of Antibacterial Activity

The antibacterial properties of nanofiber membranes against S. aureus ATCC 6536 and E. coli ATCC 35218 were determined using the modified inhibition zone test qualitative method. Nanofibrous membranes (6 mm × 6 mm) were disinfected using UV radiation for 40 min. The bacterial suspensions with a specific concentration (optical density at 600 nm = 0.06), measured using a UV–VIS spectrophotometer (Yoke 1700, China) were cultured on MHA plates. The prepared plates were then incubated at 37 °C for 24 h.

Time-kill test or bacterial colony counting method at specified time points (1, 2, 4, and 6 h) was also carried out by the AATCC test method 100-2019 to assess bacterial reduction kinetics.^27^ To prepare a 0.5 McFarland standard, bacterial strains of E. coli and S. aureus were shaken at 130 rpm for 24 h at 37 °C. The nanofiber membranes were weighed before being sterilized with UV irradiation for 40 min. Then, they were submerged in diluted bacterial suspensions (10^5^ CFU mL^–1^) for 1, 2, 4, and 6 h to assess quantitative antibacterial activity. The bacterial suspensions were then diluted to one-tenth three times to allow colony counting. The diluted suspensions were cultured on agar plates and incubated at 37 °C for 24 h. In addition to the samples, the aforementioned procedure was repeated with no samples for the control group. After counting the surviving bacterial colonies at each time point, the reduction rate of bacteria by CUR was calculated using the bacterial colony counting method described in a study.^39^ The antibacterial experiments were repeated three times.

### Biodegradability Study

The biodegradability of nanofiber membranes was studied using the natural soil burial test.^51^ Fresh soil (pH 8.5, measured using Schott pH meter, Camlab, England) was collected from the campus garden of the Public Health Faculty at Tehran University of Medical Sciences. The nanofiber membrane samples with specified dimensions (30 mm × 10 mm) were buried in soil that was kept moist by sprinkling distilled water twice weekly. Weight loss due to biodegradation was measured weekly. The samples were taken from soil and dried in an oven at 50 °C to achieve a constant weight.^52^ The test was also conducted on a commercial filter.

### Data Analysis

To determine the mean difference between groups, a one-way analysis of variance (ANOVA) followed by the Bonferroni post hoc test was used. Pearson correlation analysis also revealed a relationship between the studied variables. Statistical tests were conducted using SPSS software (SPSS Inc., USA, version 26). A p-value <0.05 was considered statistically significant.

## Results and Discussion

### Morphological Study of PVA and PVA/BA Nanofibers Loaded with CUR

FE-SEM images and diameter distributions of the prepared electrospun nanofibers loaded with CUR and BA are shown in Figure 1. The preceding research optimized the electrospinning parameters for the fabrication of neat PVA and PVA/BA nanofibers with mean diameters of 111 and 171 nm, respectively.^44^ In the present research, PVA and PVA/BA solutions were separately loaded with CUR, and the nanofibers were then obtained under the optimal electrospinning parameters listed in Table 1. As shown in Figure 1, none of the samples contained beads on the nanofibers. The presence of thicker nanofibers indicates that the stretching ability of the solutions decreases after adding CUR, as well as at higher concentrations. This can be attributed to the physicochemical properties of the solutions, which include viscosity, surface tension, and electrical conductivity. Sufficient solution viscosity is required to produce a uniform jet during electrospinning while minimizing the effects of surface tension.^53^ Increasing the solution viscosity to an appropriate level after adding CUR can inhibit the stretching of the polymeric jet during the electrospinning process, resulting in thicker, more uniform fibers.^54,55^ Additionally, CUR can alter the conductivity of the solutions, which affects the charge density on the jet surface, impacting the stretching and bending instability of the jet, which in turn influences the fiber diameter.^53^ This result is consistent with studies by Fatahian et al. (2020),^56^ and Gupta et al. (2016).^57^ Similarly, the PVA/BA/CUR nanofibers were thicker than PVA/CUR nanofibers. This result could also be attributed to the decrease in stretching of the solutions after adding BA, which likely increases the viscosity and decreases the conductivity of the solutions. While the addition of CUR could increase the diameter of PVA nanofibers, Fereydouni et al. (2021) found that the mean diameter of zein nanofibers containing nanocurcumin at comparable concentrations (5–15 wt %) decreased due to reduced solution viscosity.^58^ Differences in solution chemical properties (polymer type, concentration, solubility, solvent),^59^ and electrospinning parameters could lead to this inconsistency. According to the FE-SEM images in Figure 1, CUR concentrations of 5 and 10 wt % appear to have been thoroughly mixed in the aqueous PVA solution, as evidenced by the absence of observable CUR aggregates (Figure 1). However, aggregates appeared in some areas on the surface of the PVA/BA nanofibers containing 15 wt % CUR (C-3) (Table 2).

**Figure 1:**
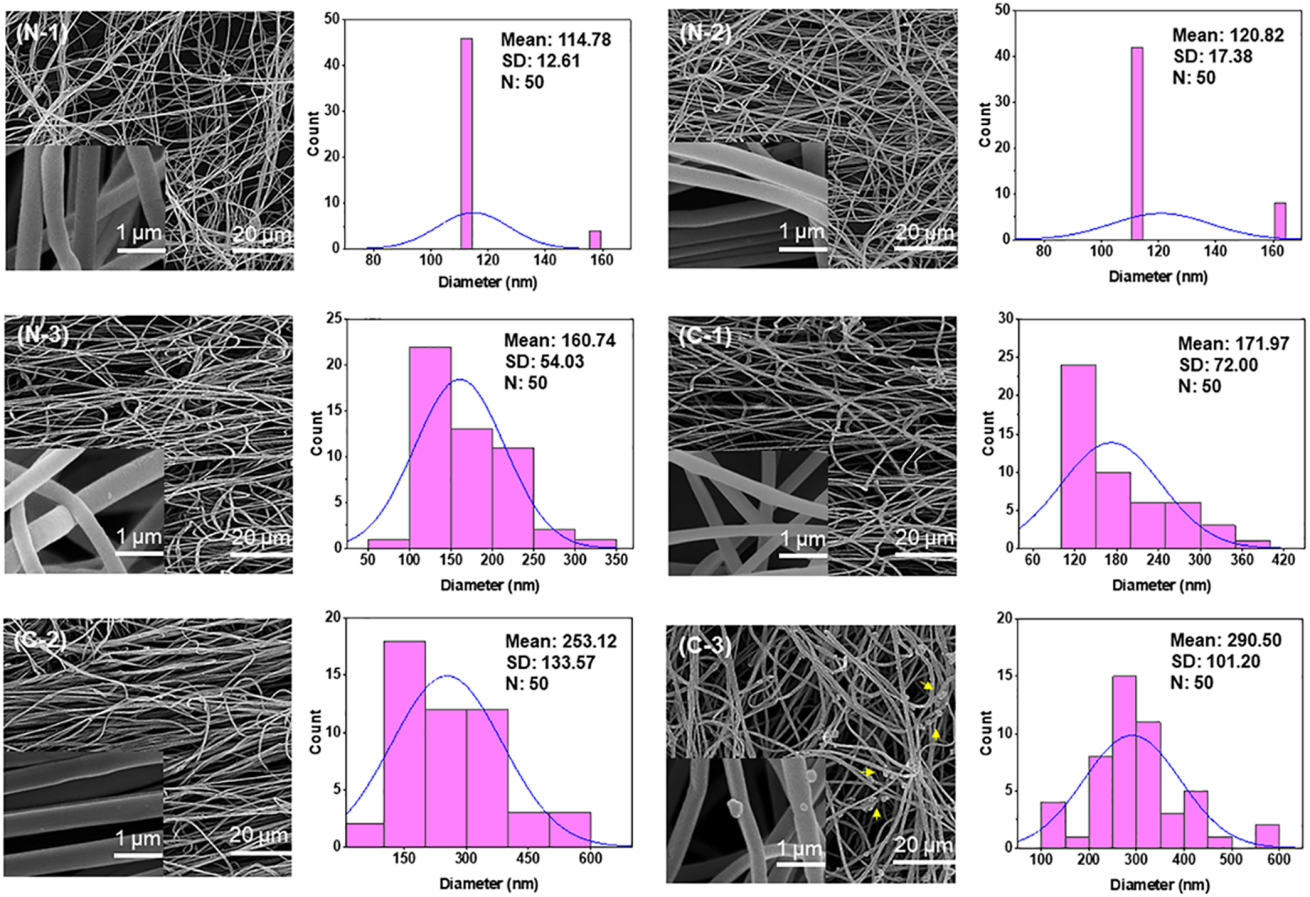
FE-SEM images, and nanofiber diameter distributions of the prepared PVA and PVA/BA electrospun membranes with different CUR concentrations from 5 to 15 wt %.

**Table 2:** Mean and SD of Physicochemical Characteristics of the Nanofiber Samples

Codes	N-1	N-2	N-3	C-1	C-2	C-3
Fiber diameter (nm)	114.8 ± 12.6	120.8 ± 17.4	160.7 ± 54.0	172.0 ± 72.0	253.1 ± 133.6	290.50 ± 101.2
Thickness (mm)	0.114 ± 0.001	0.117 ± 0.001	0.117 ± 0.002	0.136 ± 0.001	0.115 ± 0.001	0.139 ± 0.001
Packing density	0.135 ± 0.001	0.140 ± 0.000	0.140 ± 0.002	0.127 ± 0.000	0.140 ± 0.000	0.127 ± 0.001
Porosity (%)	86.5 ± 0.10	86.03 ± 0.06	86.00 ± 0.17	87.33 ± 0.06	85.97 ± 0.06	87.30 ± 0.10

### Chemical Nature of the Nanofibers

The presence of BA in the PVA/BA nanofibers was confirmed in the preceding research.^44^ Figure 2 depicts the FTIR spectra of the samples, revealing the chemical structure of CUR in the PVA/CUR nanofibers derived from sample N-3 and the PVA/BA/CUR nanofibers produced from sample C-3. The spectrum of neat CUR shows peaks at 3381, 2933, and 1234 cm^–1^, corresponding to the stretching of phenolic O–H, C–H, and C–O–C in-plane bending groups, respectively.^60,61^ Furthermore, the characteristic peaks for the carbonyl group stretching and the mixing of vibrations of the C=C and C=O stretching groups of the sample were observed at 1420 and 1628 cm^–1^, respectively. In the case of samples N-3 and C-3, the ether group of CUR shifted slightly to approximately 1242 cm^–1^. Additionally, new peaks appeared at 1725 and 1713 cm^–1^ after CUR interacted with PVA and PVA/BA, which can be assigned to the carbonyl bond.^62^

**Figure 2:**
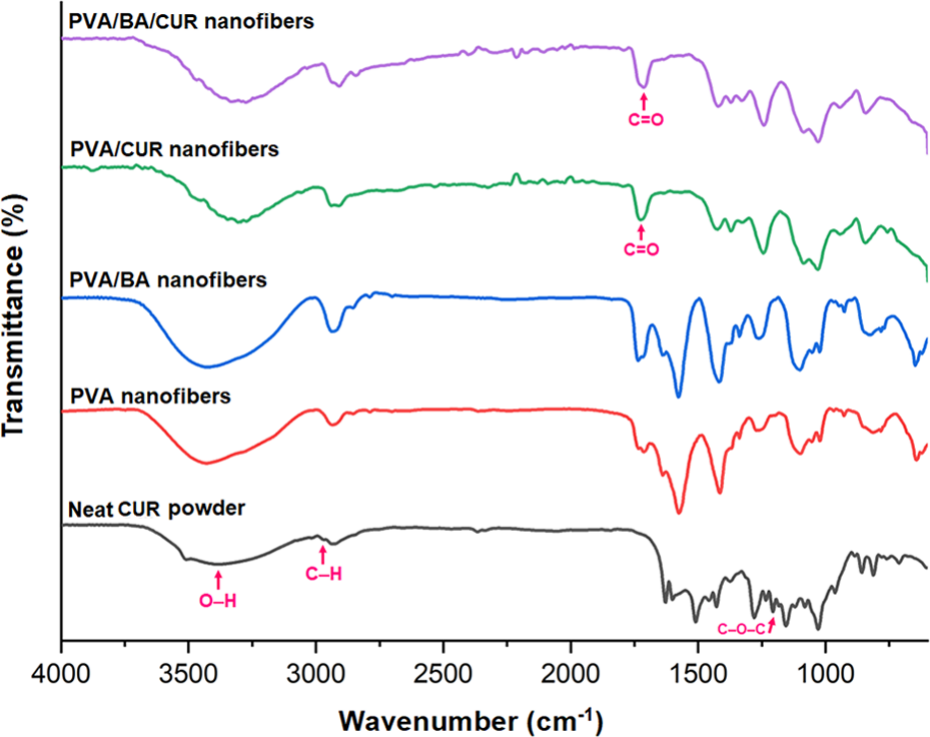
FTIR spectra of the neat CUR powder, and the nanofibers of neat PVA, PVA/CUR, PVA/BA, and PVA/BA/CUR.

### Mechanical Properties of Nanofibers

To investigate the effect of CUR on the mechanical properties of PVA and PVA/BA nanofiber membranes, tensile tests were performed on the membranes. Neat PVA and PVA/BA nanofiber membranes showed tensile strengths of 4 and 4.40 MPa, respectively.^44^ According to the results in Table 3, adding CUR to PVA and PVA/BA nanofibers increased the strength of the samples. This can be attributed to interactions between CUR and PVA molecules, which affect polymer chain entanglement and improve hydrogen bonding in the membrane structures.^63^ The tensile strength of the samples also improved with an increase in CUR concentration so that the PVA/10 wt % (*p* < 0.05) and 15 wt % CUR nanofibers (*p* < 0.05) had significantly higher tensile strength values than the PVA/5 wt % CUR nanofibers (Table 3). It was observed that the tensile strength of the PVA/BA/10 wt % CUR nanofibers was significantly higher than that of the PVA/BA/15 wt % CUR (*p* < 0.05), but this parameter did not statistically differ between the PVA/10 wt % CUR and PVA/15 wt % CUR nanofibers (*p* > 0.05). The strain and Young’s modulus of the samples of PVA/CUR and PVA/BA/CUR nanofibers differed significantly (*p* < 0.001). The highest strain and Young’s modulus were observed when the PVA and PVA/BA nanofiber membranes were loaded with 10 wt % CUR (Table 3). Additionally, the increase in CUR content first improved the tensile properties of the electrospun membranes, after which they began to reduce. This observation is similar to what was reported in another study on electrospun zein-based membrane filter containing 1 wt % nanoemulsion curcumin, where the increase in CUR concentration led to greater interaction forces between molecular chains.^64^ Furthermore, that study reported a lower tensile strength (0.72 MPa) than that of the current investigation, which could be due to the different polymer used in the membrane structure and the higher concentration of CUR in the current study, which helped create more hydrogen bonding interactions in the membrane structure.

**Table 3:** Tensile Properties of the Prepared Electrospun Nanofiber Membranes

Codes	N-1	N-2	N-3	C-1	C-2	C-3
Strength (MPa)	4.56 ± 0.10	5.36 ± 0.16^a^	5.15 ± 0.15^a^	4.77 ± 0.10	4.90 ± 0.10	4.53 ± 0.12
Strain (%)	26.88 ± 0.11	43.84 ± 0.17^b^	39.93 ± 0.13^b^	30.04 ± 0.13	33.49 ± 0.11^c^	28.86 ± 0.13^c^
Modulus (MPa)	38.78 ± 0.09	49.53 ± 0.18^b^	44.85 ± 0.13^b^	40.15 ± 0.08	41.13 ± 0.12^c^	39.29 ± 0.14^c^

a Significant difference between N-1 and the other sample group (*p* < 0.05).

b Significant difference between N-1 and the other sample group (*p* < 0.001).

c Significant difference between C-1 and the other sample group (*p* < 0.001).

### Filtration Performance of Nanofibers Against PMs

The structural properties of electrospun nanofibers make them highly appealing for filtration applications. However, these fibers are mechanically fragile, resulting in thin, delicate webs with insufficient structural integrity for practical handling and testing. As a result, nanofiber membranes are typically collected on supporting substrates to allow for handling and preserve their integrity during postprocessing.^65,66^ Nonwoven spunbond polypropylene is a popular substrate because it provides mechanical strength and flexibility while not interfering with the nanofiber layer’s performance. Figure 3a,b demonstrates the optimal filtration efficiency of nanofiber membranes and the spunbond layer alone for various particle sizes (0.3, 0.5, 1, and 3 μm) at airflows of 4.7 and 5.3 cm s^–1^. The results indicated that the spunbond layer had a filtration efficiency of about 16% for 0.3 μm particles (Figure 3a,b), and a low pressure drop (0 Pa), indicating limited intrinsic filtration capability. The nanofibers electrospun on the spunbond layer could improve the filtration efficiency for the particles compared to the substrate alone. As seen in Figure 3a,b, the filtration efficiency of the nanofiber membranes improved as particle size increased from 0.3 to 1 μm. Among the membranes, sample N-1 had slightly greater filtration efficiency for submicron particles of 0.5 and 0.3 μm, which corresponds to the most penetrating particle size (MPPS) (100% and 99.97% at 4.7 cm s^–1^; 100% and 99.99% at 5.3 cm s^–1^, respectively). The PVA/CUR nanofiber membrane achieved a higher efficiency (99.99% in N-1) for 0.3 μm particles compared to the neat PVA and PVA/BA nanofiber membranes (99.92% and 99.93%, respectively).^44^ This demonstrates that the use of CUR improved filtration efficiency. It also showed slightly higher filtration efficiency than a PVA/copper nanoparticles electrospun membrane reported in another study (99.98%),^24^ which could be attributed to the greater mean fiber diameter (410 nm) and the use of an aqueous electrospinning solution.

**Figure 3:**
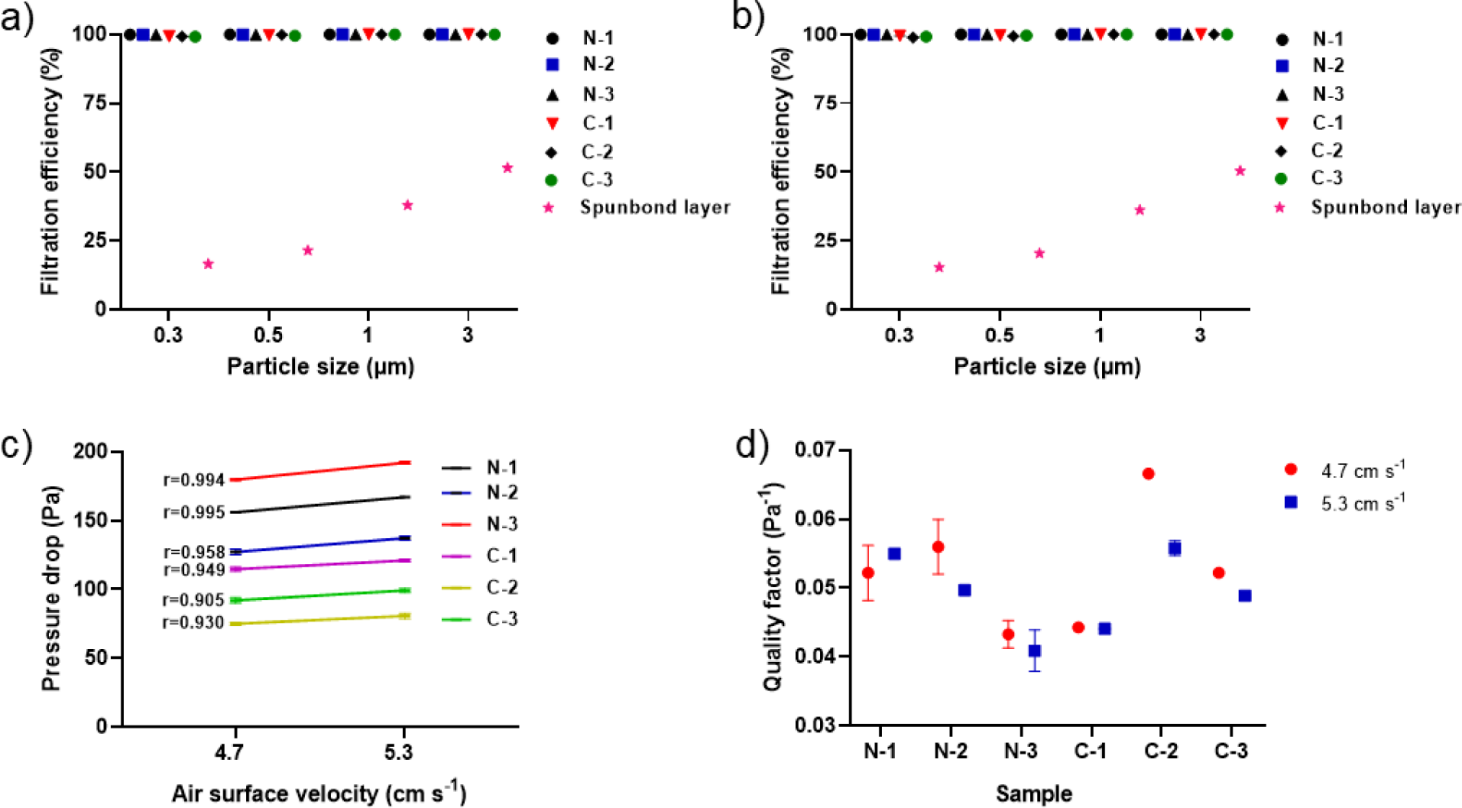
Filtration performance of the PVA and PVA/BA nanofiber membranes with different CUR concentrations, including filtration efficiency for the particle sizes of 0.3–3 μm at (a) 4.7 and (b) 5.3 cm s^–1^ airflow rates, (c) pressure drop values at two air velocity rates and correlation between the variables, (d) quality factor under the studied two air surface velocities.

The submicron particles are typically trapped via the interception mechanism, and the use of a green solvent, along with the compositions and optimal electrospinning conditions, can improve fiber morphology and effective interception,^67^ resulting in high filtration efficiency. In this study, electrospinning aqueous solutions were prepared, and nanofiber membranes were obtained using optimal instrumental conditions. As a result, sample N-1, with thinner fibers (mean diameter of 115 nm) exhibited higher filtration efficiency. The increase in CUR content from 5 to 15 wt % reduced the filtration efficiency of the PVA nanofiber samples, resulting in higher filtration efficiency for all particle sizes when the CUR content was at its lowest (5 wt %). The enhanced interception mechanism, due to the use of a aqueous solution for fiber fabrication, and the presence of CUR—the phenolic small compound capable of occupying the space between fibers and forming the hydrogen bonds with the hydroxyl groups of PVA molecules^68^—can lead to a more coherent structure of the nanofiber membrane filter. Furthermore, the PVA/CUR nanofiber membranes with thinner fibers can provide higher filtration efficiency than the PVA/BA/CUR membranes. The filtration efficiency decreased slightly in membranes with the higher CUR content of 5 wt %, with the lowest filtering ability for the MPSS at 5.3 cm s^–1^ assigned to samples C-2 and C-3 (98.91% and 99.22%, respectively). This is also likely due to the larger mean fiber diameters (290 and 253 nm, respectively), which resulted in a lower interaction between the nanofibers and the particles.^69^

Figure 3c shows the relationship between the pressure drop of the PVA/CUR and PVA/BA/CUR nanofiber membranes at various airflow velocity rates. Linear correlations exist between these two aforementioned variables in all membranes. Sample N-1, with a mean fiber diameter of 115 nm, exhibited the greatest optimal filtration efficiency for the 0.3 μm particles, resulting in a pressure drop of 167 Pa at 5.3 cm s^–1^. The pressure drop amount was higher than 150 Pa for neat PVA and PVA/BA nanofiber membranes^44^ due to the increase in mean fiber diameters after adding CUR. Furthermore, the obtained pressure drop is much lower than that reported for other PVA-based electrospun membrane (518 Pa at 5 cm s^–1^) with a somewhat similar efficiency (99.98%),^24^ which could be influenced by relatively thicker fibers (410 nm) and a larger pore size (about 1800 nm).

On the other hand, the prepared nanofiber membranes demonstrated relatively low pressure drop values due to their structural properties (Table 2). Among the membrane filters, sample N-3, which had a higher CUR content, exhibited the highest pressure drop (179 Pa at 4.7 cm s^–1^, and 192 Pa at 5.3 cm s^–1^), most likely due to structural properties like mean fiber diameter and packing density (161 nm and 0.14 g m^–2^, respectively). Furthermore, sample C-2 with a low thickness (0.115 mm) achieved during the optimal electrospinning time and a relatively higher mean fiber diameter (253 nm), demonstrated the lowest pressure drop (75 Pa at 4.7 cm s^–1^, and 81 Pa at 5.3 cm s^–1^) (Figure 3c).

In addition to the diameter and thickness of the filter, air surface velocity influences the air pressure drop.^70^ Figure 3c depicts the shift in pressure drop values as a function of air surface velocity. As the air velocity rose from 4.7 to 5.3 cm s^–1^, the air resistance of the nanofiber membranes elevated linearly in all the membrane filters, ranging between 10–13 Pa for the PVA/CUR nanofiber membranes and 3–7 Pa for the PVA/BA/CUR nanofiber membranes. This also indicates that some PVA/BA/CUR membrane filters (samples C-2 and C-3) showed relatively lower filtration efficiency for fine particles and less energy consumption for passing air, which could be a result of the increased fiber diameter and thickness of the membranes. Also, CUR aggregates may have an effect on the filtration performance of sample C-3.

To evaluate the filtration performance of the prepared nanofiber membranes containing CUR, the quality factor was utilized^67^ for the MPPS of 0.3 μm. As shown in Figure 3d, the quality factor of the membranes improved as the CUR content increased from 5 to 10 wt %, but reduced with higher CUR content (15 wt %), which can be related to the increase in pressure drop for CUR content greater than 10 wt %. In this study, sample C-2 exhibited the greatest quality factor (0.067 Pa^–1^ at 4.7 cm s^–1^, and 0.056 Pa^–1^ at 5.3 cm s^–1^) due to the lowest pressure drop value (Figure 3c), which is significantly greater than 0.016 Pa^–1^ reported in another study on an antibacterial PVA membrane with a similar thickness of 0.115 mm.^[24]^ A low quality factor could be due to the membrane’s structural properties, which would result in a much higher pressure drop (above 500 Pa). A commercial high-efficiency particulate air (HEPA) filter can provide 99.99% filtration efficiency for neutralized NaCl particles of 0.3 μm with a pressure drop of 488 Pa at 5.3 cm s^–1^.^44^ In this study, the optimal filtration efficiency of sample *N*-1 (99.99%) is comparable to that of the commercial filter. Furthermore, this nanofiber membrane, with a relatively low basis weight (18.11 g m^–2^), could serve as an effective fine particle filter with significantly lower air resistance than a commercial filter, as well as biodegradability (see the biodegradability section).

### Biofiltration Efficiency of Nanofibers

The BFE of the nanofiber membranes was tested using a bacterial model of S. aureus aerosolized at a constant air flow rate and pressure. The BFE values of neat PVA and PVA/BA nanofiber membranes were almost similar (around 79% and 78%, respectively). The addition of CUR improved BFE in nanofibers (Table 4). Consequently, the comparison between neat PVA membrane and N-1 exhibits that adding CUR has the potential to increase BFE by 16%. This improvement could be due to changes in the structural properties of these membranes after adding CUR, as well as enhanced antibacterial activities (see the next section). Among the nanofiber PVA/CUR membranes, the lower CUR content was associated with higher BFE (approximately 92% for sample N-1), whereas higher CUR contents (i.e., 10 and 15 wt %) resulted in a decrease in BFE. According to Table 4, the observed increase in BFE% when increasing the CUR content from sample C-1 to C-3 (from 94.94% to 96.74%) was statistically significant (*p* < 0.05). Furthermore, the BFE of the PVA/BA/CUR nanofiber membranes was higher than that of PVA/CUR nanofibers membranes (*p* < 0.05), so that the highest BFE was obtained from sample C-3 at approximately 97%, exceeding the minimum BFE requirement of 95% according to the ASTM F2101-19 standard.^50^ The antibacterial and morphological properties of sample C-3 may have an impact on this result. In other words, sample C-3, with its higher bacterial inhibition ability (see the next section), greater fiber diameter (around 290 nm), and thicker membrane (0.130 mm), could be effective in high trapping bioaerosols.

**Table 4:** Results of the Biofiltration Test of the Tested Samples

Samples/codes	Colony count	BFE (%)
N-1	15.33 ± 2.51	91.68
N-2	29.33 ± 1.53	84.10
N-3	32 ± 1	82.64
C-1	9.33 ± 1.53	94.94
C-2	9.33 ± 0.57	94.94
C-3	6 ± 1	96.74
Spunbond layer	80 ± 2	56.60

The filtration efficiency for 3 μm NaCl particles in the nanofiber membranes is comparable to that of BFEs due to their similar particle size distribution (MPS of 3 μm). All membranes achieved 100% filtration efficiency for 3 μm particles, while the membranes containing CUR had BFE values ranging from 82.64% to 96.74%. This demonstrates how the type of particles used to test the membrane can impact its filtration efficiency. According to the results, nanofiber membranes may provide better protection against chemical particles than biological ones. The higher filtration efficiency could be attributed to factors such as solid/wet particles, air surface velocity, particle charge, and particle size distribution.^71^ In the present research, we considered a constant particle size distribution (3 μm) and a specific velocity rate (4.7 cm s^–1^) to compare the filtration efficiencies of the samples established for particles of different natures. As a result, the filtration efficiency was most likely influenced by the behavior of neutralized and dry NaCl aerosols compared to negatively charged and liquid-phase S. aureus aerosols.

### Antibacterial Properties of Nanofibers

Curcumin has been used as an antibacterial agent in several studies to produce nanofiber membranes from various polymer solutions using the electrospinning method.^38,58,72^ As previously stated, nanoformulation of curcumin has demonstrated significantly higher aqueous solubility than curcumin^40,73^ due to its larger surface area.^74^ As a result, this study produced electrospun nanofiber membranes loaded with varying concentrations of CUR obtained from the aqueous solutions, and the antibacterial activity of the samples was investigated using two methods: inhibition zone and time-kill methods against E. coli, and S. aureus. The results of the qualitative inhibition zone method revealed that the neat PVA and PVA/BA nanofiber samples exhibited no antibacterial activity against the two bacterial strains. Furthermore, the nanofiber membranes containing 5 to 15 wt % CUR did not show any clear inhibition zone on S. aureus, whereas the majority of the samples, including N-1, N-2, N-3, C-1, and C-3 showed antibacterial activity against E. coli with the zone of growth inhibition of 7.0 ± 1.0, 7.0 ± 1.0, 11.3 ± 0.6, 7.3 ± 0.6, and 10.3 ± 1.5 mm, respectively (Figure 4). Although several studies demonstrated antibacterial properties of curcumin,^75,76^ a study found no antibacterial activity on electrospun zein-based nanofibers containing 5 to 15 v/v% nanoemulsion curcumin against each of the bacterial strains of Pseudomonas aeruginosa, E. coli, and S. aureus with diluted suspensions (10^6^ CFU mL^–1^) using inhibition zone test method, most likely due to the low amounts of potential antibacterial agent available in the nanofibers and type of the antibacterial test applied.^58^ In contrast, the current study found antibacterial effect of the nanofibers containing CUR against E. coli and not against S. aureus. This result could be explained by the structure of the bacterial strains’ cellular walls. Since E. coli‘s peptidoglycan wall is thinner than that of another strain,^77^ CUR may be able to penetrate the bacterial cell more easily, leading to its death.

**Figure 4:**
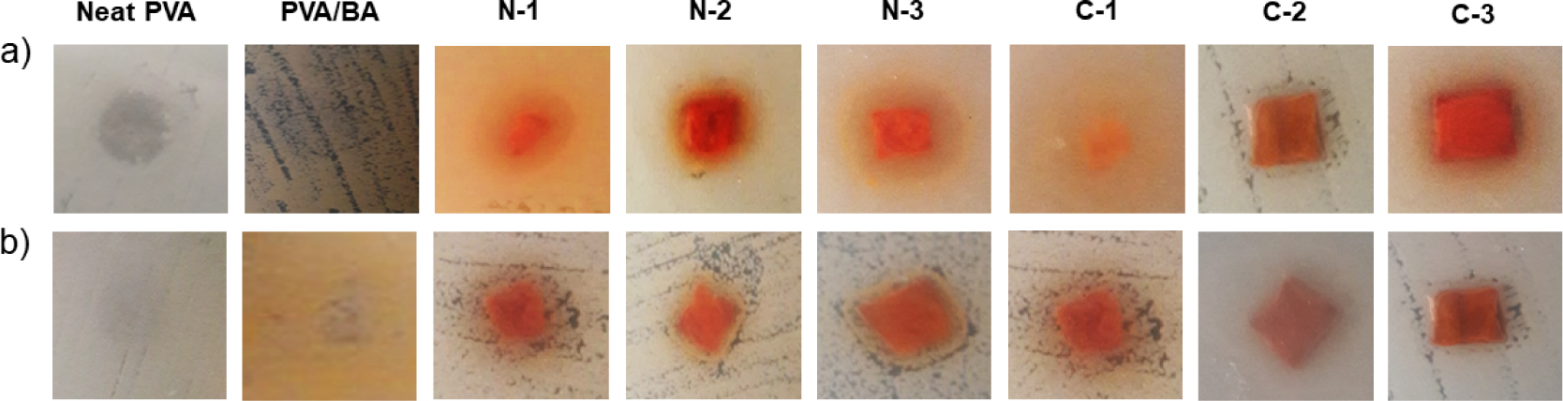
Antibacterial activity of nanofiber samples using the inhibition zone method against (a) E. coli and (b) S. aureus.

Another antibacterial test that was used to quantitatively evaluate the efficacy of the nanofiber membranes loaded with CUR was the time-kill method. As shown in Figure 5, the highest E. coli reduction rate was observed within 2 h of exposure for most of the samples, including N-2, N-3, and C-2 but not for N-1, C-1, and C-3. The highest antibacterial performance levels of samples N-1 and C-1 were within 1 and 4 h exposure to the E. coli solution which is likely due to the lower CUR content in the samples. Additionally, the time point of 6 h indicated the highest reduction rate for both bacteria, with E. coli and S. aureus reducing around 92% and 63% in C-3, respectively. This finding could have been influenced by aggregations of the CUR on the fibers in C-3 (according to SEM results) and the sample pH. Curcumin is a typical Bronsted–Lowry acid, which protonates and deprotonates in response to pH changes. Furthermore, curcumin is unstable at pH above 7 due to reduced stability of its multiple hydroxyl anions.^78^ A study demonstrated pH-responsive curcumin release, with no release from electrospun PVA/graphene/oxide-silver/curcumin nanofibers at pH 7.4, but an increase in release at an acidic microenvironment (pH 5.4).^79^ In the current study, all of the electrospun samples had a pH of less than 7, which it indicates the stability of CUR in the electrospun solutions. However, sample C-3 showed the highest bacterial reduction rate, which could be attributed to the more controlled release of CUR^39^ from the sample, which had a more acidic electrospun solution (pH 5.36) than the others. Furthermore, although samples N-3 and C-3 contained the same CUR content (15 wt %), the presence of BA contributed to a higher acidity for the solution of C-3 than that of N-3 (pH 5.36 vs 6.15, respectively), resulting in higher antibacterial performance of C-3. The samples exposed to E. coli showed significant antibacterial activity due to the greater reduction rate of this bacterial strain than that of S. aureus. This result, which is consistent with the inhibition zone test results of the current study, demonstrates that curcumin works against different bacteria by causing different cell membrane structural damage.^75^ In the PVA/CUR membranes, sample N-3 showed the greatest reduction for E. coli and S. aureus after 2 h (around 80% and 58%, respectively). On the other hand, N-1 had the lowest reduction rate for E. coli and S. aureus after 2 h (around 49% and 54%, respectively) (Figure 5). These results may be influenced by the greater amount of CUR (15 wt %) compared to the other concentrations. In general, the antibacterial activity of CUR-loaded electrospun nanofiber membrane may be affected by factors such as the amount of CUR,^58^ the kind of antibacterial test, the solvent system (e.g., water, dimethyl sulfoxide, ethanol), which influences curcumin solubility,^80^ the pH of the electrospinning solution, which impacting the stability of curcumin anions,^79^ and the method used to synthesize nanoformulation of curcumin.^60^

**Figure 5:**
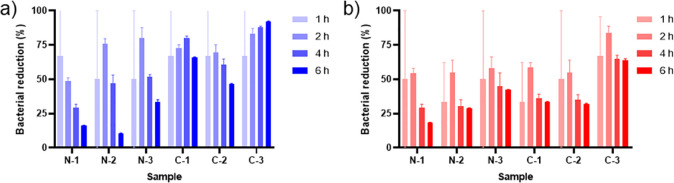
Antibacterial activity efficacy of the PVA and PVA/BA nanofiber membranes with different CUR concentrations against (a) E. coli and (b) S. aureus at different time intervals.

### Biodegradability of Nanofibers

As mentioned previously, the effect of curcumin on the biodegradability of PVA nanofibers was studied by burying the samples in natural soil. Biodegradation in soil is facilitated by the activity of microorganisms such as bacteria and fungi.^81^ It was found that after being buried in soil, the samples became smaller, harder, and wrinkled. The biodegradability rates of neat PVA and PVA/BA nanofiber membranes were 11.92% and 4.77%, respectively, at the end of the fourth week. All of the prepared nanofiber membranes containing CUR degraded in the natural soil after 4 weeks, whereas the weight of the commercial filter remained constant. As shown in Figure 6, increasing the CUR concentration from 5 to 10 wt % resulted in an insignificant change in weight loss for N-2 compared to N-1 after 4 weeks of soil burial. The samples with the CUR concentration of 10%, i.e., N-2 and C-2, degraded more than the other samples by the end of the fourth week (around 4.5% and 3.9%, respectively). It could be due to an increase in hydrophilicity and the sample’s ability to absorb more soil moisture.^82^ Furthermore, the PVA/BA/CUR nanofibers lost less weight than the PVA/CUR nanofibers due to cross-linking PVA with BA. The PVA/BA nanofibers containing 10 wt % CUR (C-2) lost 3.9% of their weight by the end of the fourth week, which was approximately 2.5 times more than C-1 (Figure 6). In contrast, the PVA/BA sample with CUR concentration above 10 wt % (C-3) showed a lower biodegradability rate than C-2, thus, the reduction in weight loss of sample C-3 was around 2% whereas it was 3.9% for sample C-2 after 4 weeks, likely due to aggregates of CUR on the fibers and higher antibacterial activity of C-3, which might have impacted soil microorganisms and also reduced the hydrophilicity of C-3,^83^ resulting in less weight loss percentages for C-3. Overall, the biodegradability rate of PVA nanofibers decreased as CUR and BA were added to the membrane structure, which could be due to their nature even though they are biobased. This could be because as CUR and BA form strong hydrogen bonds with PVA. Hence, BA and CUR may decrease the biodegradability rate of the PVA nanofibers due to the decrease in soil moisture absorption, and the antibacterial activity of CUR. Furthermore, increasing the concentration of CUR to a certain level can improve the absorption of soil moisture and increase the degradability of the network structure due to soil microbes. While the current study maintained consistent moisture conditions by regularly sprinkling distilled water during the test period, the effect of varying water content on the degradation rate of the nanofibers would be investigated in future research.

**Figure 6:**
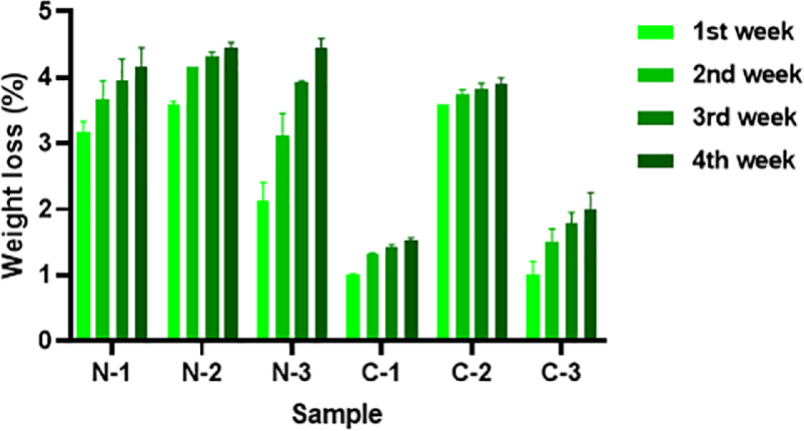
Weight loss of the PVA and PVA/BA nanofiber membranes with different CUR concentrations in the natural soil test.

## Conclusions

In the current study, PVA and PVA/BA nanofibers with different concentrations of CUR including 5, 10, and 15 wt % were successfully electrospun from aqueous solutions. In optimal electrospinning conditions, the nanofiber membranes remained bead-free. Morphological analyses revealed that using a higher concentration of CUR increased mean fiber diameter. CUR aggregates were observed in some areas on the surface of the C-3 sample. FTIR analysis revealed the presence of curcumin in the nanofiber membranes. The addition of CUR increased the average diameter of the fibers. Thicker fibers were also produced as the CUR concentration increased. The addition of CUR to the PVA and PVA/BA nanofibers improved the samples’ mechanical properties. Furthermore, CUR-loaded nanofiber membranes demonstrated superior filterability for both fine aerosols and bioaerosols. The membranes with the highest filtration efficiency were obtained from PVA nanofibers with lower CUR concentrations. It could also produce significantly less pressure drop than a commercial HEPA-grade filter. Biofiltration data revealed that increasing the CUR concentration from 5 to 10 wt % in PVA/BA nanofibers could provide high filtration efficiency for bacterial particles. The nanofiber membranes containing CUR showed no inhibition zone against S. aureus, while demonstrating more effective antibacterial performance against E. coli compared to against S. aureus according to the time-kill method. Thus, the evaluation of the antibacterial performance of the samples using the quantitative method could provide more comprehensive results. Also, with the highest antibacterial activity observed on PVA/BA nanofibers containing a higher CUR content, i.e., 15 wt % against both the bacteria. Finally, biodegradability data showed that the nanofibers containing 10 wt % CUR degraded more in natural soil than other CUR-loaded membranes. As a result, the findings indicate that biobased nanofiber membranes containing CUR and BA electrospun from aqueous solutions, with their antibacterial performance, could be promising candidates for air filtration applications to effectively control fine particulate matter pollution and protect against biological particles.
